# Masking a fish's detection of environmental stimuli: application to improving downstream migration at river infrastructure

**DOI:** 10.1111/jfb.13812

**Published:** 2018-12-21

**Authors:** James R. Kerr, Paul S. Kemp

**Affiliations:** ^1^ International Centre for Ecohydraulics Research, Faculty of Engineering and Physical Sciences, Department of Civil, Maritime and Environmental Engineering University of Southampton Southampton UK

**Keywords:** brown trout, fish behaviour, bypass entrance, sensory ecology, signal detection theory, velocity gradient

## Abstract

According to Signal Detection Theory, the ability to detect a stimulus (discriminability, *d'*) is inversely related to the magnitude of internal and external noise. In this study, downstream moving brown trout *Salmo trutta* were used to investigate whether external hydrodynamic noise (in this case turbulence) could mask a signal that induced an unwanted response, such as rejecting accelerating velocity gradients commonly encountered at entrances to fish bypass channels. *S. trutta* behaviour was quantified in the absence (control) or presence of an accelerating velocity gradient created by an unconstricted or constricted channel, respectively, under two levels (low and high) of background turbulent kinetic energy (hydrodynamic noise). Experiments were conducted in an indoor recirculating flume in the dark and a range of passage metrics were quantified. Under the control condition, most (*ca*. 91%) *S. trutta* passed, usually oriented downstream (67%), with minimal delay (median 0.13 min). In comparison, fewer *S. trutta* (*ca*. 43%) passed under constricted conditions, they tended to orient facing into the flow (*ca*. 64%) and delay was greater (median > 20 min). When viewed from a coarse‐scale perspective, discriminability of the velocity gradient was lower when turbulence was high suggesting masking of the signal occurred. However, the resulting increase in the percentage of fish that passed, decrease in time to pass and reduction in the distance at which *S. trutta* reacted (switched orientation) was subtle and non‐significant. Despite the mixed results obtained, the use of masking to manipulate an animal's perception of environmental stimuli as a fisheries management tool is conceptually valid and the results of this experiment present a useful stepping stone for future research.

## 1 INTRODUCTION

For downstream migrating fish, high levels of mortality can occur during passage through dangerous routes at anthropogenic infrastructure (*e.g*., turbines and spillways; Calles *et al*., 2010; Stokesbury & Dadswell, 1991; Taylor & Kynard, 1985). Direct causes of mortality include blade strike, shear, cavitation, rapid decompression (barotrauma), gas supersaturation and mechanical damage; indirect causes include stress, disorientation and elevated susceptibility to predation and disease (Kemp, 2016). To mitigate for these adverse effects, physical or behavioural screens are used to guide downstream migrating fish away from dangerous routes to more desirable bypass channels (Larinier, 2008). However, the efficacy of these systems is often low (Calles *et al*., 2012; Kynard & O’Leary, 1993), with fish avoiding conditions encountered at bypass entrances (Kynard & Buerkett, 1997) resulting in delayed migration (Ovidio *et al*., 2016) and increased probability of passage through hazardous routes (Johnson *et al*., 2000).

Accelerating velocity gradients are common at bypass entrances (Haro *et al*., 1998). Numerous studies have analysed fish behaviour as they encounter velocity gradients (Enders *et al*., 2009, 2012; Haro *et al*., 1998; Kemp *et al*., 2005, 2006, 2008; Russon & Kemp, 2011; Vowles & Kemp, 2012; Vowles *et al*., 2014), but relatively little attention has been directed at reducing rejection rates; among the exceptions are Haro *et al*. (1998) and Vowles and Kemp (2012). On encountering velocity gradients, downstream moving juvenile salmonids typically switch orientation to face the flow and actively reject the near‐field hydrodynamic transition by swimming back upstream; for example, Atlantic salmon smolts *Salmo salar* L. 1758 (Ovidio *et al*., 2016); Pacific salmon smolts *Oncorhynchus* spp. (Kemp *et al*., 2005); brown trout *Salmo trutta* L. 1758 (Vowles & Kemp, 2012). Partial success in reducing the rejection rate at a bypass entrance was achieved by altering the intake design and reducing the rate of acceleration (*i.e*., the signal strength) that fish experience as they pass (Haro *et al*., 1998). However, physical alterations that sufficiently reduce the rate of acceleration at bypass intakes may not always be possible. Vowles and Kemp (2012) tested the effect of localized illumination on rejection of a velocity gradient and found that avoidance and delay exhibited by *S. trutta* increased rather than decreased when multimodal stimuli were present. A better understanding of the sensory ecology and behavioural response of fish in relation to hydraulic stimuli is required to improve passage efficacy through bypass systems if the adverse effects of anthropogenic barriers are to be mitigated.

Psychophysics, a branch of psychology that explores the relationship between physical stimuli and perception, has been successfully utilized for over a century in the fields of sensory ecology and animal behaviour (Akre & Johnsen, 2014; Fechner, 1860; Graham, 1934). Signal detection theory (SDT), a key concept within psychophysics, considers the relationship between the magnitude and perceived intensity of a stimulus (signal) and the ability to discern between the signal and noise (discriminability, *d'*; Kemp *et al*., 2012). Kemp *et al*. (2012) utilized SDT to analyse the behavioural response of fish to different velocity gradients and highlighted its usefulness as a tool for understanding, quantifying and potentially manipulating how fish perceive and respond to sensory stimulus. An important premise within SDT is that *d'* is inversely related to the magnitude of internal and external noise. This masking effect, defined as a change in the probability of perceiving a signal in the presence of a second stimulus (Gelfand, 2010), has been observed for a diverse range of species in relation to multiple sensory modalities; *e.g*., sound (Lohr *et al*., 2003; Wollerman & Wiley, 2002), vibration (Wu & Elias, 2014), vision (Woo *et al*., 2009) and hydrodynamic signal detection (Bassett *et al*., 2006; Engelmann *et al*., 2002; Kröther *et al*., 2002). However, to the best of our knowledge, masking has never been used to limit the detection of a signal for conservation purposes, such as the protection of fish at river infrastructure.

Building on the results of Kemp *et al*. (2012), the aim of this study was to assess whether increased turbulence reduced the discriminability of an accelerating velocity gradient by downstream moving *S. trutta* in an experimental flume in the absence of visual cues. This was experimentally tested by assessing the behaviour of *S. trutta* in the absence (control) or presence of an accelerating velocity gradient created by an unconstricted and constricted channel, respectively, under high or low turbulent kinetic energy (TKE). The results were analysed in respect to: 1) coarse scale behaviours, the percentage of fish that approached and passed and time to pass; 2) fine scale behaviours, fish orientation, nature of response (orientation switch or rejection) and response location; 3) SDT, *d'* and a measure of response bias (response criterion), calculated from both a coarse and fine‐scale perspective. It was predicted that *S. trutta* would be less likely to reject the accelerating velocity gradient when background levels of TKE were high, resulting in increased numbers passing through the constriction with reduced delay.

## 2 METHODOLOGY

### 2.1 Experimental setup

**Figure 1:**
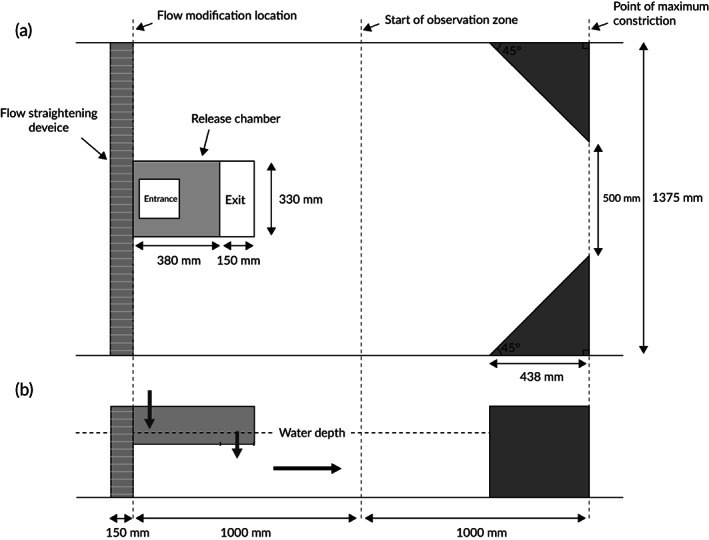
(a) Top and (b) side‐view schematic of the experimental area with velocity gradient present under low levels of hydrodynamic noise (TL, treatment low). (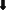)**,** The entrance and exit of the release chamber; (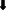)**,** direction of bulk flow; (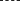), the flow modification location, the start of the observation zone, and the point of maximum constriction

Experiments were conducted in a 2 m section of an indoor recirculating flume (21.40 m long, 1.38 m wide and 0.60 m deep) at the International Centre for Ecohydraulics Research (ICER), University of Southampton, UK (Figure 1). Under the treatment condition, an accelerating velocity gradient was created by gradually constricting the channel to 36.4% of its width using triangular wooden baffles aligned with their upstream face at 45 ° to the flow direction (Figure 1). Fish response in the presence (treatment) and absence (control) of the constriction was assessed under low or high turbulence (TKE). Turbulence was manipulated by screening the upstream extent of the experimental area with either a flow straightening device (low TKE) or a large diameter grid (high TKE). The flow straightener consisted of a 100 mm thick polycarbonate screen with elongated tubular (7 mm diameter) porosity. The mesh grid consisted of orthogonally aligned and equally spaced (120 mm) wooden batons (33 mm wide × 18 mm thick). The test conditions created were control‐low (CL), control‐high (CH), treatment‐low (TL) and treatment‐high (TH).

Discharge was maintained constant (0.09 m^3^ s^−1^) during all treatments. A similar free stream velocity (*U*_*f*_, across channel mean measured 1 m upstream from the constriction point 150 mm above the flume floor) among treatments (Table 1) was controlled by adjusting an overshot weir at the downstream end of the channel. Water depth (*d*) was slightly deeper when the channel constriction was present (treatment = 288 mm, control = 280 mm; Table 1). A surface mounted release chamber (mesh cage 0.53 m long, 0.33 m wide, 0.15 m deep), located 1.62 m upstream of the point of maximum constriction, allowed fish to volitionally enter the experimental area. The release chamber protruded 60 mm below the surface and fish entered the experimental area through an orifice (0.15 m long, 0.33 m wide) located in its base (Figure 1). Fish were prevented from exiting the experimental area upstream by the flow straightening device or by a fine wire mesh (12 mm diameter) attached to the upstream face of the grid. Fish could freely pass downstream beyond the point of maximum constriction. Experiments were conducted at night. A 1.0 m section upstream of the point of maximum constriction, the observation zone (Figure 1), was illuminated with infrared light and monitored using a low‐light overhead CCTV camera.

**Table 1:** Conditions encountered by *Salmo trutta* in experimental trials conducted to assess their behavioural response to a velocity gradient under different levels of hydrodynamic noise

Velocity gradient	Hydrodynamic noise	Treatment notation	TKE (mean ± SD; J m^−3^)	*U*_*f*_ (mean ± SD; m s^−1^)	Depth (mm)	Replicates	Temperature (mean ± SD; °C)	Fork length (mean ± SD; mm)
Control	Low	CL	0.23 ± 0.00	0.27 ± 0.01	280	22	10.46 ± 0.00	153.1 ± 12.1
	High	CH	1.52 ± 0.18	0.26 ± 0.00	280	21	10.45 ± 0.04	147.1 ± 11.8
Treatment	Low	TL	0.23 ± 0.03	0.26 ± 0.01	288	21	10.68 ± 0.05	147.3 ± 10.6
	High	TH	1.61 ± 0.07	0.26 ± 0.01	288	21	11.24 ± 0.07	146.1 ± 11.7

TKE, Mean turbulent kinetic energy recorded within the observation zone.

*U*_*f*_, Mean across‐channel velocity measured 1.0 m upstream of the point of maximum constriction (150 mm depth).

Water velocities were measured for each treatment (*n* = 132, 132, 507 and 521 in CL, CH, TL and TH, respectively) using an acoustic doppler velocimeter (ADV; Vectrino, Nortek, AS; www.nortekgroup.com; sample frequency 50 Hz, sample volume 0.05 cm^3^, record length 90 s) 150 mm above the bed (0.53 *d*). Hydraulics conditions were measured at a single depth as vertical variation in the flow was assumed to be minimal due to the lateral constriction of the channel and vertically uniform spacing of the mesh grid and flow straightener. Velocity data was collected at 0.53 *d* rather than 0.60 *d* (the depth that best approximates average vertical flow velocity; Herschy, 1995), as noise at this depth, probably acoustic reflection from the flume bed, interfered with ADV performance. Fewer hydraulic measurements were recorded in the control treatments as the flow environment was more homogeneous. Mean velocity (*U*, m s^−1^; Equation (1)) and TKE (J m^−3^; Equation 2) were calculated from the filtered (3D cross correlation filter; Cea *et al*., 2007) ADV data:

(1) $$U={\left({\bar{u}}^{2}+{\bar{v}}^{2}+{\bar{w}}^{2}\right)}^{0.5}$$

(2) $$\mathrm{TKE}=0.5\rho \left(\bar{{u^{\prime }}^{2}}+\bar{{v^{\prime }}^{2}}+\bar{{w^{\prime }}^{2}}\right)$$

where *u*, *v* and *w* are the instantaneous velocity values corresponding to the *x*, *y* and *z* spatial coordinates, overbar and prime denote time‐averaged and deviation from mean, respectively, and *ρ* is density (kg m^−3^). Within the hydrodynamically mapped region (horizontal‐longitudinal plane within which velocity was measured, 150 mm from the flume bed), conditions were linearly interpolated (1 mm resolution). Outside of the mapped region (30 mm area adjacent to the flume walls) boundary layer conditions were calculated (1 mm resolution) by fitting a third order polynomial to the adjacent interpolated points and extrapolating to the boundaries.

**Figure 2:**
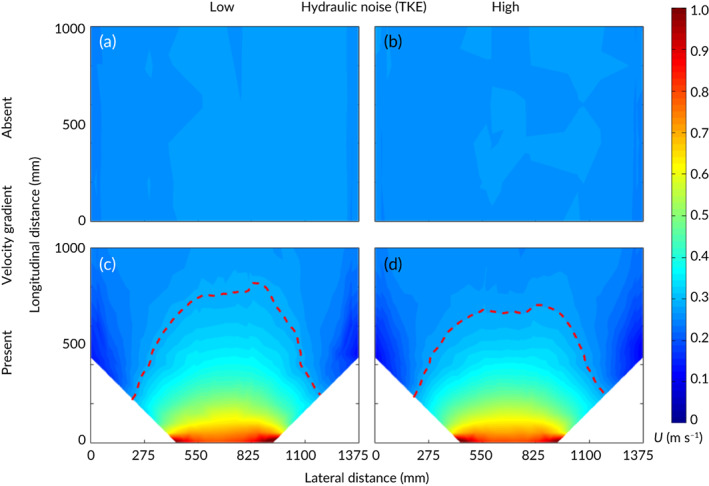
Colour intensity plots of velocity (m s^−1^) in the observation zone under the four treatments created in the absence (a, b) and presence (c, d) of a constriction under low (a, c) or high (b, d) turbulent kinetic energy. The behavioural response of *Salmo trutta* was quantified under the four treatments. (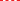), Delineation of the extent of the velocity gradient (acceleration zone)

**Figure 3:**
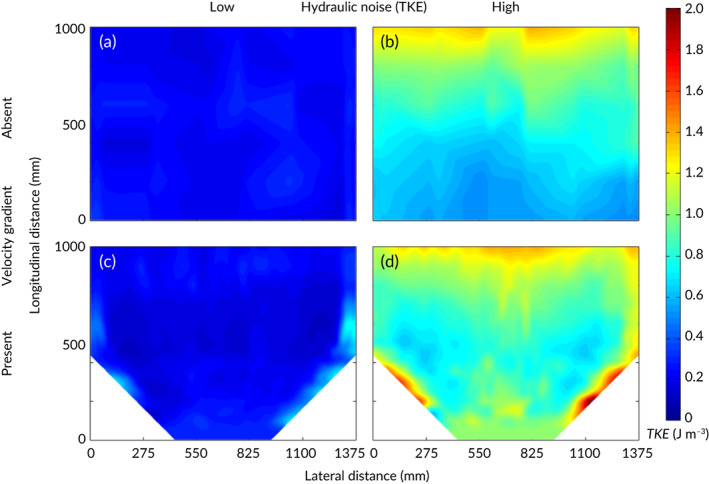
Colour intensity plots of turbulent kinetic energy (TKE – J m^−3^) in the observation zone under the four treatments created in the absence (a, b) and presence (c, d) of a constriction under low (a, c) or high (b, d) hydrodynamic noise. The behavioural response of *Salmo trutta* was quantified under the four treatments

**Figure 4:**
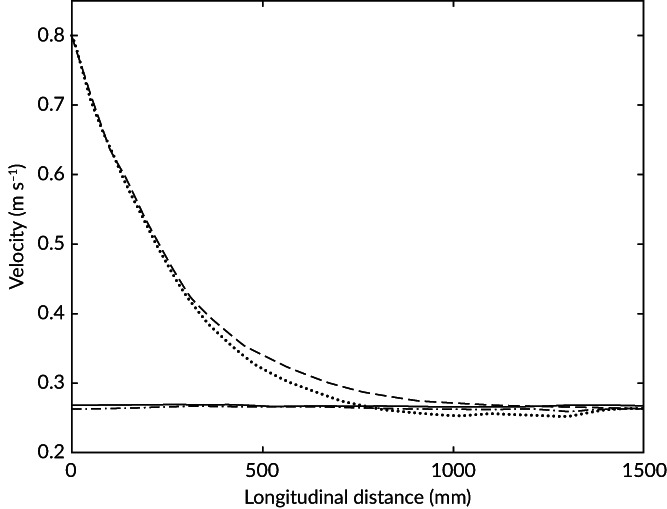
Flow velocity along the central longitudinal axis of the flume, measured from the point of maximum constriction (or equivalent under control conditions), under the four treatments in which the behavioural response of brown trout *Salmo trutta* was quantified. CH: velocity gradient absent, high hydrodynamic noise; CL: velocity gradient absent, low hydrodynamic noise; TH: Velocity gradient present, high hydrodynamic noise; TL: velocity gradient present, low hydrodynamic noise. (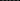) TH, (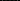) TL, (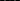) CL, and (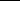) CH

In the constricted and unconstricted channel, velocities ranged from *ca*. 0.13–1.03 and *ca*. 0.24–0.29 m s^−1^, respectively (Table 1 and Figure 2). Mean TKE recorded in the observation zone was *ca*. 0.23 and *ca*. 1.55 J m^−3^ under low and high hydrodynamic noise treatments, respectively (Table 1 and Figure 3). The longitudinal spatial flow velocity increase (m s^−1^ m^−1^) was non‐linear and similar under both low and high TKE (Figure 4). In addition, the area where velocity was at least 10% greater than free stream, a proxy for the spatial extent of the velocity gradient (hereafter referred to as the acceleration zone), was similar under both low and high TKE (Figure 2).

### 2.2 Experimental procedure

*Salmo trutta* (*n* = 85, mean ± SD fork length *L*_F_ = 148 ± 12 mm, range = 128–169 mm) were transported in an aerated transport tank from Leckford Estate Trout Fishery, Hampshire, UK (51° 07′ 55.9′′ N, 1° 28′ 31.9′′ W), to the ICER facility on 1 October, 2012. They were held in filtered and aerated 3,000 l external holding tanks (pH 7.8, ammonia 0 ppm, nitrite 0 ppm, nitrate < 40 ppm, 50% weekly water change) at ambient temperature (mean ± SD *T* = 7.76 ± 1.40°C).

Eighty‐five trials were conducted between 1 and 4 November 2012 (flume temperature: mean ± SD *T* = 10.67 ± 0.27 °C; Table 1). Prior to each trial fish were placed in a porous container in the flume and allowed to acclimatize for at least 1 h. Each trial commenced when a single fish was placed in the release chamber and ended when it had either passed downstream beyond the point of maximum constriction (or equivalent location under control conditions) or after 20 min. Each *S. trutta* was used only once and weighed (g) and measured (*L*_F_, mm) at the end of each trial. After experimentation fish were humanly killed by immersion in an overdose of anaesthetic (2‐phenoxyethanol: 1 ml l^−1^) followed by confirmation of death by permanent cessation of the circulatory system.

### 2.3 Fish behaviour

**Figure 5:**
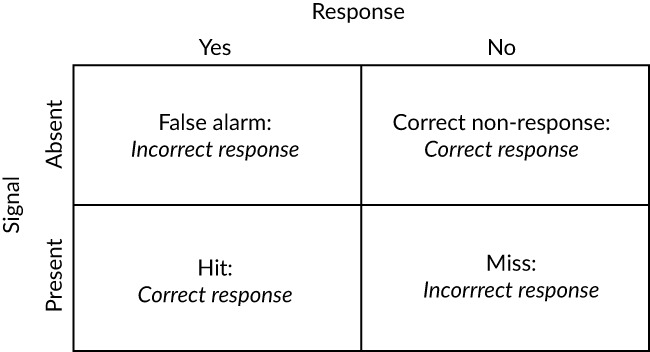
The four potential signal‐response outcomes that may occur in the presence or absence of a specific environmental stimulus. In this study, the signal was the velocity gradient and the response was downstream passage (coarse‐scale assessment) or displaying avoidance behaviour (fine‐scale assessment) during a trial

The results were analysed in respect to: 1) coarse scale behaviours, percent approached, percent passed and time to pass; 2) fine scale behaviours, approach orientation and pass orientation (positive or negative rheotaxis), nature of response (orientation switch or rejection) and distance of initial response (*R*_*d*_, mm) from the constriction; 3) SDT, discriminability (*d'*) and response criterion (*c*), from both a coarse and fine scale perspective (Table 2). The metrics *d'* and *c* are measures of whether an organism can detect a stimulus and the level at which an internal response results in the exhibition of a behaviour, respectively. The advantage of using SDT to assess animal behaviour is that it accounts for response bias, *i.e*., the tendency for an animal to react as though a signal is present even if one is not. The two SDT metrics were assessed by calculating the percentage of fish that correctly or incorrectly passed downstream (coarse scale perspective) or displayed a behavioural response (fine‐scale perspective) when the flume was either constricted or unconstricted (Table 2). Based on an assumption that fish should avoid an accelerating velocity gradient (signal), under the coarse‐scale assessment fish were considered to have responded correctly if they failed to pass downstream under constricted conditions (hit; Figure 5) and incorrectly if they failed to pass downstream under control conditions (miss; Figure 5). When considering the fine scale perspective, fish were considered to have responded correctly if they displayed either an orientation switch or rejection (or both) when the flume was constricted (hit; Figure 5) or incorrectly if they displayed such a behavioural response under control conditions (false alarm; Figure 5).

**Table 2:** Definition of passage metrics obtained and the statistical tests used to assess the behaviour of *Salmo trutta* as they passed downstream through an unconstricted or constricted section of a recirculating flume under low or high levels of background turbulent kinetic energy (TKE)

Category	Metric	Definition		Statistical test
	Percent approached	Percentage of fish that entered the observation zone – 1 m section upstream of the point of maximum constriction (Figure 1)		Pearson's *X* ^*2*^ *‐*tests
Coarse scale behaviours	Percent passed	Percentage of fish that passed downstream beyond the point of maximum constriction		Pearson's *X* ^*2*^ *‐*tests
	Time to pass	Time between when the fish approached and passed		Kaplan–Meier product‐limit estimator and the log rank (mantel‐cox) statistic (*X* ^*2*^_*mc*_)
	Approach and pass orientation	Percentage of fish that exhibited positive (facing into flow) or negative (aligned with flow) rheotaxis as they approached or passed		Pearson's *X* ^*2*^ *‐*tests
Fine scale behaviours	Nature of response	Percentage of fish that displayed either: a) Orientation switch ‐ change from negative to positive rheotaxis or vice versa, or b) Rejection ‐ cessation of downstream movement followed by upstream movement of at least half a body length		Pearson's *X* ^*2*^ *‐*tests
	Distance of initial response *(R*_*d*_ *)*	Distance from the point of maximum constriction at which fish displayed their initial response. Extrapolated from the closest point (fish head or tail position) to the point of maximum constriction. Mean *R*_*d*_ values only calculated for trials when the flume was constricted and for responses (orientation switch or rejection) that occurred when the fish was at least partly within the *acceleration zone* (Figure 2)		Student t‐tests and robust bootstrapped (n = 2000) bias corrected and accelerated (BCa) confidence intervals (CI) (95%)^a^
Signal detection theory	Discriminability *(d’)*	*d*^′^ = *Z*_*H*_ − *Z*_*FA*_	Where Z_H_ and Z_FA_ are the standard deviation units (Z scores of the unit normal Gaussian distribution) of the probability of a Hit and false alarm Fine scale assessment: Hit: Exhibited a behavioural response in the presence of the velocity gradient *False alarm:* Exhibited a behavioural response in the absence of the velocity gradient Coarse scale assessment*:* Hit: Did not pass downstream in the presence of the velocity gradient False alarm: Did not pass downstream in the absence of the velocity gradient	N/A
	Response criterion *(c)*	$c=-\frac{Z_{H}+Z_{\mathrm{FA}}}{2}$		

a Efron and Tibshirani (1993).

The metric *d*' (Table 2) is a measure, in standard deviation units (*Z*‐scores), of the separation between the means of the noise and signal‐plus‐noise frequency distributions (assumed to be normally distributed with similar variance) and can be calculated from the hit rate (HR) and false alarm rate (FAR; Kemp *et al*., 2012). *d'* values of 1 and 2 are equivalent to one and two standard deviations of separation, respectively, with higher values representing higher levels of signal discriminability. The metric *c* (Table 2) is a measure, in standard deviation units, of the distance of the response criterion from being unbiased (equal probability of a false alarm or a miss; Stanislaw & Todorov, 1999; Wickens, 2002). If the response criterion is unbiased, *c* has a value of 0. Negative values of *c* signify a bias toward responding yes whereas positive values signify a bias toward responding no.

The influence of the following variables, constriction (present or absent), turbulence (high or low) and approach orientation (positive or negative rheotaxis, where applicable) on each metric was assessed using appropriate statistical tests (Table 2). Time‐to‐pass was assessed using time to event analysis (Singer & Willet, 2003) (Table 2), a method that provides unbiased estimates compared with conventional statistical techniques by including fish that fail to pass downstream (right‐censored individuals) in a probability function (reported here as the cumulative probability of passage) at any given time (Castro‐Santos & Haro, 2003). Fish orientation and location data during each trial was acquired from the video footage by tracking the head and tail position using LoggerPro 3.8.2 (Vernier Software and Technology; www.vernier.com). Data manipulation was undertaken using Matlab 7.10.0.499 (www.mathworks.com), statistical analysis using SPSS v.20.0.0 (IBM; www.ibm.com) and figures produced using Matlab, SigmaPlot 12.5.038 (www.sigmaplot.co.uk) and Microsoft Publisher 14.0.7106.5003 (www.microsoft.com).

The research was reviewed and sanctioned by the University of Southampton Ethical Review Board.

## 3 RESULTS

### 3.1 Coarse scale behaviours

**Figure 6:**
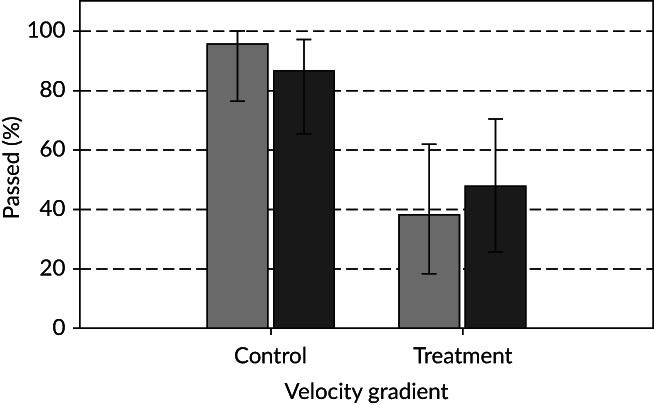
Mean (±95% CI) percentage of all *Salmo trutta* that passed downstream when velocity gradient was absent with low (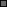) hydrodynamic noise (CL, *n* = 22), velocity gradient was absent with high (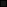) hydrodynamic noise (CH, *n* = 21), velocity gradient was present with low hydrodynamic noise (TL, *n* = 21) and velocity gradient was present with high hydrodynamic noise (TH, *n* = 21)

All fish exited the release chamber within 5.83 min (median duration 0.15 min). The percentage of fish that approached was not influenced by constriction or by turbulence, with only one *S. trutta* failing to do so under the CL treatment. The percentage of fish that passed was influenced by constriction (*X* ^*2*^(1) = 22.014, *P* < 0.001), with more *S. trutta* passing downstream under control *v*. treatment conditions (combined average: 90.7 *v*. 42.9%) (Figure 6). Under the constricted treatment, more fish passed under high (47.6%) compared with low (38.1%) TKE, but this difference was not statistically significant (Figure 6). The percentage of fish that passed was also not influenced by approach orientation.

**Figure 7:**
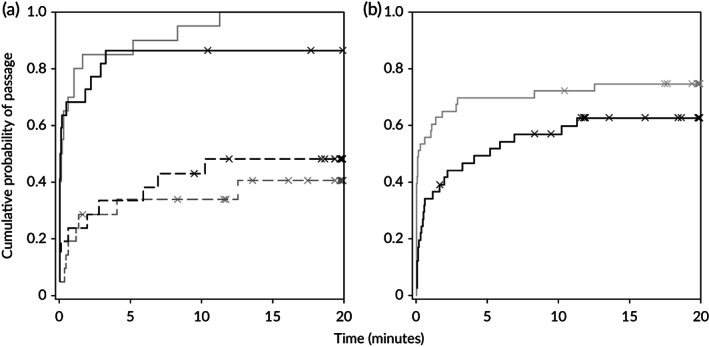
(a) Cumulative probability of passing downstream against time for *Salmo trutta* in the presence (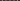, 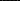) and absence (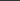, 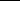) of a channel constriction under low (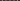, 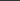) and high (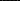, 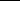) turbulent kinetic energy (TKE). (b) Cumulative probability of passing downstream against time for *S. trutta* that entered the observational zone facing upstream (positive approach rheotaxis, 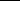) and downstream (negative approach rheotaxis, 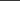); aggregated data for constricted and control treatments. X, instances of right‐censored data

Time‐to‐pass was influenced by constriction (*X* ^*2*^_*MC*_ (1) = 31.599, *P* < 0.001; Figure 7(a)) and approach orientation (*X* ^*2*^_*MC*_(1) = 4.707, *P* < 0.05; Figure 7(b)). Delay tended to be longer under the constricted treatment (median > 20 min *v*. median 0.13 min; Figure 7(a)) and for individuals that exhibited positive rheotaxis when they approached (median 5.18 min *v*. median 0.16 min; Figure 7(b)). Under the constricted treatment there was a slight increase in the cumulative probability of passage after *ca*. 6 min under high compared with low TKE (Figure 7(a)) but this did not translate to a significant influence of turbulence on time‐to‐pass.

### 3.2 Fine‐scale behaviours

Approach orientation and pass orientation were influenced by constriction (*X* ^*2*^(1) = 5.765, *P* < 0.05 and X^2^(1) = 6.510, *P* < 0.05, respectively), but not turbulence. *Salmo trutta* tended to exhibit positive rheotaxis whilst they approached and passed when the flume was constricted (61.9 and 69.2%, respectively), but exhibit negative rheotaxis when they approached and passed under the control (64.3 and 66.7%, respectively; data combined from both low and high turbulence treatments). Under control conditions, pass orientation was influenced by approach orientation (FET: *P* < 0.05), with *S. trutta* more likely to pass downstream facing the same direction as they approached. Pass orientation was not influenced by approach orientation when the flume was constricted.

Within the observation zone the majority of *S. trutta* (*ca*. 64%) displayed an orientation switch at least once (Table 3). The percentage of fish that displayed an orientation switch was not influenced by approach orientation, turbulence, or constriction (Table 3). The percentage of *S. trutta* that showed at least one rejection was influenced by constriction (*X* ^*2*^(1) = 16.045, *P* < 0.001), but not by approach orientation or by turbulence, with a higher percentage doing so under the constricted treatment (85.7 *v*. 44.2%; Table 3).

**Table 3:** Percentage of *Salmo trutta* facing the flow (positive rheotaxis) and that exhibited an orientation switch or rejection at least once as they approached and passed downstream through a flume in which a constriction was either present (treatment) or absent (control) under low or high turbulent kinetic energy (TKE)

Treatment^a^	Orientation (% positive)		Orientation switch (%)	Rejection (%)
	Approach	Passage		
Control‐low	40.0	45.0	65.0	55.0
Control‐high	31.8	15.8	54.5	36.4
Treatment‐low	66.7	75.0	66.7	90.5
Treatment‐high	57.1	60.0	71.4	81.0

a Control and treatment relate to velocity gradient; low and high relate to hydrodynamic noise (Table 1).

**Figure 8:**
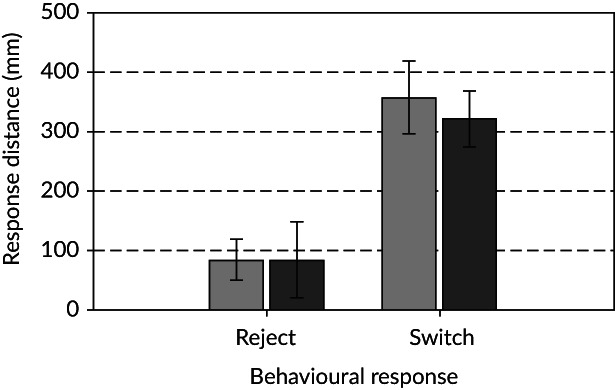
Mean (±95% CI) response distance at which *Salmo trutta* responded (rejection or orientation switch) to a velocity gradient created by a channel constriction under low (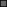) and high (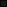) turbulent kinetic energy

**Figure 9:**
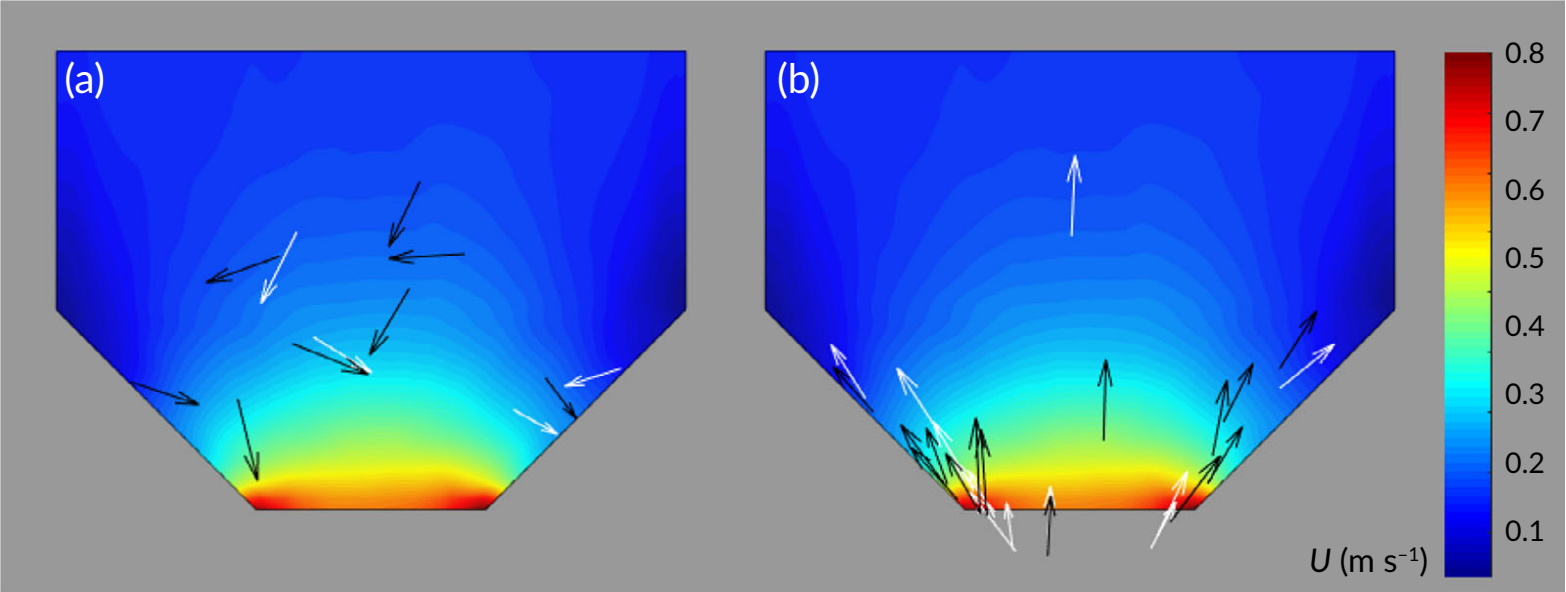
Location of initial (a) orientation switch and (b) rejection by *Salmo trutta* when the velocity gradient was present under low (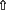) and high (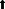) turbulent kinetic energy superimposed onto colour intensity plots of flow velocity (*U*). Arrow head indicates head position of *S. trutta*

Orientation switches tended to occur further away from the point of maximum constriction (*R*_*D*_ ≈ 345 mm) than rejections *(R*_*D*_ ≈ 85 mm; *μ* diff.: +262 mm, BCa CI: [154, 374], *t*(36) = 4.492, *P* < 0.001; Figures 8, 9). *Salmo trutta* appeared to exhibit orientation switches closer to the constriction under high (*R*_*D*_ = 337 mm) *v*. low (*R*_*D*_ = 360 mm) TKE, but the influence of turbulence on *R*_*D*_ was not significant for either orientation switches or rejections (Figures 8, 9).

### 3.3 Signal detection theory

**Figure 10:**
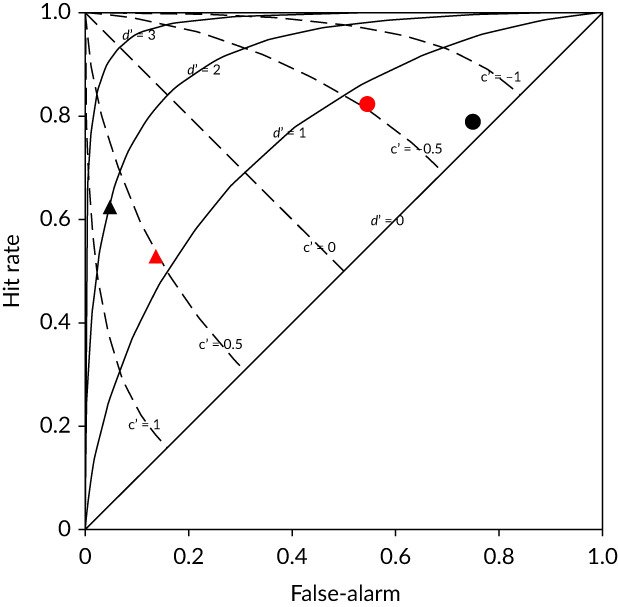
Receiver‐operating characteristics plot of hit rate against false‐alarm rate for coarse (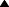 **,** 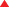) and fine (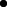 **,** 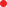) scale assessment of the behavioural responses of *Salmo trutta* to a velocity gradient under low (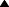 **,** 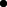) and high (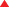 **,** 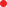) turbulent kinetic energy. Reference discriminability (*d’* = 0, 1, 2, 3; 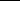) and response criterion (*c* = −1, −0.5, 0, 0.5, 1; − – –). Increases in *d’* represent greater signal discriminability and increases in *c* represent greater bias towards responding

Coarse scale signal discriminability was lower under high (*d' =* 1.16) compared with low (*d'* = 1.97) TKE (Figure 10). The coarse scale response criterion was positive and similar under low and high TKE (*c* = 0.68 and 0.52, respectively), indicating a general bias to passing downstream (Figure 10). Counterintuitively, fine scale signal discriminability was higher under high (*d'* = 0.81) compared with low (*d'* = 0.13) TKE (Figure 10). The fine scale response criterion was negative and similar under low and high TKE (*c* = −0.75 and −0.52, respectively), indicating a general bias towards exhibiting a behavioural response (Figure 10).

## 4 DISCUSSION

This study investigated the potential to use turbulence to reduce the discriminability of an accelerating velocity gradient by downstream moving *S. trutta* in an experimental flume in the absence of visual cues. During experimentation, *S. trutta* displayed behaviours typical of downstream moving salmonids, facing downstream in an unconstricted channel, but positively oriented towards the flow and displaying frequent rejections when encountering a velocity gradient, (Haro et al., 1998; Kemp et al., 2005; 2006; Enders et al., 2009b). Vowles *et al*. (2014) directly linked avoidance behaviours (*e.g*., rejections) with increased delay and distance travelled before downstream passage. Similar links were evident in this study, with a lower proportion of passes and increased delay when the channel was constricted. When viewed from a coarse‐scale perspective, signal discriminability was lower when turbulence was high suggesting masking of the signal occurred. However, the resulting increase in the percentage of fish that passed, decrease in time‐to‐pass and reduction in the distance at which *S. trutta* reacted (switched orientation) was subtle and non‐significant. In addition, when viewed from a fine‐scale perspective, signal discriminability was counterintuitively higher when turbulence increased suggesting masking of the signal did not occur. The results of this study support the growing body of evidence that velocity gradients can cause downstream moving fish to be delayed at anthropogenic infrastructure, places where they can suffer elevated risk of predation (Garcia de Leaniz, 2008) and highlight the need for effective mitigation measures to be developed. However, further work is required to assess whether turbulence can be used to effectively reduce discriminability of an accelerating velocity gradient.

Trout exhibited a negative response criterion when assessment was undertaken on fine scale behaviours, indicating that they were biased towards frequently expressing orientation switches and rejections even when the flume was not constricted. There was no clear reason as to why these behaviours were frequently exhibited. The hatchery fish used in this study displayed similar behaviours to those expressed by wild fish as they encounter a velocity gradient, such as orientation switches and rejections (Enders *et al*., 2009; Haro *et al*., 1998; Kemp *et al*., 2005, 2006). However, differences in the velocity gradient response thresholds of Chinook salmon *Oncorhynchus tshawytscha* (Walbaum 1792) (Enders *et al*., 2009) *v*. *S. trutta* (Vowles & Kemp, 2012) and in the rejection rates at a bypass entrance of Atlantic salmon smolts *v*. American shad *Alosa sapidissima* (Wilson 1811) (Haro *et al*., 1998), highlight the variability of these behaviours between species. It is possible that there is also variability in how readily these behaviours are exhibited between hatchery and wild fish and this warrants further investigation.

In this study, increased hydrodynamics noise only had a small and non‐significant influence on key passage metrics. It is likely that either the signal was overly intense or the noise level was not high enough for effective masking to occur for all *S. trutta* (i.e. the signal to noise ratio was too high). Further reductions in discriminability, by reducing the abruptness of the velocity gradient or using greater intensities of turbulence, may be needed before biologically relevant shifts in passage efficiency occur. High levels of turbulence occur naturally in river systems (TKE > 20 J m^−3^ in the wake of pebble clusters in a shallow river; Lacey & Roy, 2007) and there is scope for higher levels to be used to try to mask hydrodynamic signals. However, turbulence rapidly dissipates after generation and inducing sufficiently high levels at an accelerating velocity gradient without physically interfering with fish movement may be difficult. The use of grids with greater bar width and spacing to generate larger scales of turbulence that permeate downstream further (Mohsen & LaRue, 1990) or using boundary roughness prior to and directly at the intake (Raupach *et al*., 1991) may provide a solution. Alternatively, other stimuli (*e.g*., sound, electricity or other hydrodynamic noise sources) might be employed to provide masking effects that reduce rejection rates. However, careful consideration of fish sensory systems is required to predict what the uni or multimodal effect of these stimulus sources might be (Vowles *et al*., 2014).

The mechanosensory lateral‐line system has the appropriate anatomical distribution and physiological properties to identify regional differences in flow over different parts of a fish's body (Montgomery *et al*., 2000) and it probably plays a key role in how fish sense and respond to velocity gradients. The inner ear can also provide information on changes in flow by detecting whole‐body accelerations (Pavlov & Tjurjukov, 1995; Sand & Karlsen, 2000) and fish may be able to gain additional information on the presence of a velocity gradient via this sensory system. It is possible that the inner ear plays a greater role in the discrimination of a velocity gradient under high compared with low levels of background hydrodynamic noise, as the relative sensitivity of the lateral line to regional flow differences diminishes (*i.e*., masking occurs). The contribution of the inner ear or the lateral line to detecting flow variations is likely to depend on species, stage of development and characteristics of the flow stimulus and warrants further investigation.

The findings of this study further highlight the importance of fish perception and behaviour in relation to hydraulic conditions encountered at downstream bypass facilities. Although, at a coarse scale, signal discriminability was lower when turbulence was high, suggesting masking may have occurred, high levels of hydrodynamic noise only resulted in a small and non‐significant increase in the percentage of fish that passed, decrease in time to pass and reduction in the distance at which *S. trutta* reacted (switched orientation) to the velocity gradient. It is possible that the reduction in discriminability was not great enough to significantly improve passage or that alternative sensory information (*e.g*., from the inner ear) enabled fish to adequately detect the velocity gradient, despite effective masking of the signal to the mechanosensory lateral line. Further reductions in discriminability might be produced if higher levels of background hydrodynamic noise are utilized in future experiments. Despite mixed results, the use of masking to manipulate an animal's perception of stimuli in an anthropogenically modified environment is conceptually valid and the results of this experiment present a stepping stone for future research.
